# Comparison of oscillometric blood pressure measurement by two clinical monitors: Datex Ohmeda GE S/5 and Criticare 8100E nGenuity

**DOI:** 10.4103/0019-5049.79873

**Published:** 2011

**Authors:** Harihar V Hegde, Rajashekar R Mudaraddi, Vijay G Yaliwal, P Raghavendra Rao

**Affiliations:** Department of Anaesthesiology, SDM College of Medical Sciences and Hospital, Sattur, Dharwad, India

## INTRODUCTION

Non-invasive blood pressure (NIBP) is an indirect method of measuring blood pressure (BP).[1] NIBP monitors use the principle of oscillometry and most of the available monitors measure BP upon *deflation* of the cuff. The aim of our study was to compare the accuracy of BP measurements obtained by Datex Ohmeda GE S/5 (Monitor-1, measures BP upon *deflation*) and Criticare 8100E nGenuity (Monitor-2, measures BP upon *inflation*). The secondary outcome measure was to compare the time taken by these monitors to obtain BP measurement.

## METHODS

After obtaining institutional ethical committee approval and informed consent, patients of either sex of 18-60 years, presenting for pre-anaesthetic evaluation for elective surgery, were included. Age, sex, height, weight and body mass index (BMI) were recorded. Patients with arrhythmia, BMI >25 or <18.5 kg/m^2^, patients in whom Korotkoff sounds could not be heard, and patients with history of hypertension and diabetes, and pregnant patientswere excluded.

After a rest of 5 minutes, the following four sequential same-arm NIBP measurements (systolic, diastolic and mean BP) were obtained in supine position by tying the same size cuff to the upper arm. The first and second readings were obtained by two anaesthesiologists (blinded observers) using standard sphygmomanometer by auscultatory method. The third and fourth readings were obtained by Monitor-1 and Monitor-2, respectively. A minimum of 3 minutes gap was maintained between any two measurements.

### ComfortCuff^™^ technology

Criticare 1800E nGenuity uses ComfortCuff^™^ technology (through personal communication, Criticare Systems, Inc., Waukesha, WI, USA) to determine NIBP which detects volume displacements within the artery and senses pressure variations within the BP cuff during *inflation*. The maximum cuff inflation rate is 15 mmHg/sec, with inflation limits to 300 mmHg in adult, 300 mmHg in paediatric and 150 mmHg in neonatal modes. Cuff pressure is allowed to remain above 30 mmHg for a maximum of 2 minutes. The cuff then rapidly deflates. This device has been clinically tested as per the requirements of EN 1060 and AAMI SP-10.

## RESULTS

A total of 160 BP recordings [four sets of measurements from each of the 40 patients (M/F = 24/16)] obtained were analyzed. BP values were compared using repeated measures analysis of variance (ANOVA) and a *P* value <0.05 was considered significant. Demographic data were normally distributed [Table 1]. There was no significant difference between the BP measurements by the two observers, and the Monitor-1 and observers [Table 2]. Although the systolic and diastolic BP measurements by Monitor-2 were higher compared to both the observers, the difference was not significant. Mean BP (mmHg) obtained by Monitor-2 (97.8 ± 8.5, mean ± SD) was significantly higher (*P*< 0.001) compared to both Observer-1 (88.6 ± 8.8) and Observer-2 (89.4 ± 7.5). The time taken (sec) for measurement by Monitor-2 (21.9 ± 2.3) was significantly lower (*P*< 0.001) compared to Monitor-1 (25.8 ± 2.3).

**Table 1:** Demographic data

Parameter	N = 40
Age (years)	37.5 ± 11.7
Sex (M/F) (n)	24/16
Height (cm)	162.4 ± 8.5
Weight (kg)	59.3 ± 7.5
Body mass index (kg/m2)	22.4 ± 1.7

Values as mean ± SD

**Table 2:** Blood pressure measurements and time taken for measurement (N = 40)

Blood pressure (mmHg)	Observer-1	Observer-2	Monitor-1	Monitor-2
Systolic	120.4 ± 9.5	121.6 ± 9.2	118.3 ± 10.0	123.1 ± 10.3
Diastolic	73.3 ± 10.0	74.0 ± 8.4	71.8 ± 7.1	77.5 ± 7.9
Mean	88.6 ± 8.8	89.4 ± 7.5	89.3 ± 7.5	97.9 ± 8.5*
Time taken for measurement (sec)			25.8 ± 2.3	21.9 ± 2.3§

Values as mean ± SD; Monitor-1: Datex Ohmeda GE S/5, Monitor-2: Criticare 8100E nGenuity

* *P* < 0.001 compared to Observer-1 and Observer-2

§ *P* < 0.001 compared to Monitor-1

## DISCUSSION

Accurate measurement of BP is essential to classify individuals, to ascertain blood pressure–related risk, and to guide management in various clinical settings.[2] All NIBP monitors in clinical use should be tested for accuracy. The protocol developed by the Association for the Advancement of Medical Instrumentation (AAMI) requires testing of a device against two trained human observers in 85 subjects. Recently, an International Protocol for validation of blood pressure measuring devices has been formulated.[3] However, the fact that a device passed a validation test does not mean that it will provide accurate readings in all patients.[4] The protocol of this study differs from those mentioned above, although we are not sure of the impact of this difference on the final outcome of this study.

Our study showed that although both the monitors work on the principle of oscillometry, significant difference exists between the measurements. Our study did not involve extremes of age or blood pressures where NIBP monitors are known to be less accurate.[5,6] Although the systolic, diastolic and mean BP recorded by Criticare 8100E nGenuity were higher, only mean BP was statistically significant. The mean BP is a calculated parameter in manual recordings, whereas it is the measured one in case of monitors using oscillometry. Therefore, there is no “standard” to compare the mean BP. The protocols for validating monitors[3] do not involve mean BP, whereas systolic and diastolic BP are used for comparison. Mean BP is an important parameter based on which many therapeutic decisions are made in patients under anaesthesia as well as in intensive care units. We suggest that the future protocols validating monitors should give considerations to the mean BP as well.

Criticare 8100E nGenuity required significantly less time to obtain BP measurement. This is because the recording is obtained upon inflation of the cuff, whereas time taken by monitors obtaining BP upon deflation includes rapid inflation followed by deflation of the cuff. The ComfortCuff^™^ technology may be associated with better patient comfort because of the lesser time required to obtain a BP recording and the lower peak inflation pressure even though this aspect was not assessed in the present study.

## CONCLUSION

Criticare 8100E nGenuity and Datex Ohmeda GE S/5 were equally accurate in measuring systolic and diastolic BP, while the former recorded significantly higher mean BP and was faster.
